# Editorial: Molecular targets in oncological and hematological disease management: innovations in precision medicine

**DOI:** 10.3389/fphar.2024.1494396

**Published:** 2024-09-20

**Authors:** Adrian Bogdan Tigu, Gregory Wiedman, Stefan Eugen Szedlacsek

**Affiliations:** ^1^ Department of Translational Medicine, Institute of Medical Research and Life Sciences - MedFuture, “Iuliu Haţieganu” University of Medicine and Pharmacy, Cluj-Napoca, Romania; ^2^ Department of Chemistry and Biochemistry, Seton Hall University, South Orange, NJ, United States; ^3^ Department of Enzymology, Institute of Biochemistry of the Romanian Academy, Bucharest, Romania

**Keywords:** targeted therapy, precision medicine, molecular diagnosis, innovations, nanomedicine

Targeted therapies represent a significant advancement in medicine, particularly in treating complex diseases such as cancer. These therapies, designed to specifically target biological mechanisms driving disease, can alter pathways that exacerbate disease progression, effectively slowing or halting tumor growth. Unlike traditional therapies that broadly affect both healthy and cancerous cells, targeted therapies minimize collateral damage by focusing on specific molecular triggers. This precision leads to superior outcomes while reducing adverse effects and toxicity (Buongervino et al., 2021; Fu et al., 2022; Zatovicova et al., 2022).

By providing a more specific and focused intervention, targeted therapies lead to improved treatment outcomes while reducing the risks of adverse effects and morbidity. Traditional medicines tend to affect a broader range of systems, often leading to more negative side effects (Canova et al., 2023; Disselhorst and Baas, 2020; Lohiya et al., 2023; Pralea et al., 2024). In contrast, targeted therapies focus on disease-related molecules, keeping healthy cells unexposed and minimizing patient toxicity.

Recent advances in precision medicine have accelerated the development of therapies targeting key genetic mutations or molecular abnormalities driving tumor growth (Zafar et al., 2024; Zhang et al., 2024). Personalized treatments designed according to each tumor’s unique DNA profile offer improved efficacy and reduced toxicity (Dailah et al., 2024; Guo et al., 2024; Liu et al., 2024; Tomuleasa et al., 2024). This Research Topic explores therapeutic agents that target disease-associated molecules, investigating strategies to alter molecular pathways responsible for disease progression and resistance to treatment.

Shafiei et al. investigated silibinin-loaded magnetic niosomal nanoparticles (MNNPs) for colorectal cancer treatment. Their findings demonstrated a significant increase in the cytotoxic effects of silibinin on cancer cells while sparing healthy cells. This study illustrates the growing importance of nanoparticle-based drug delivery systems in precision oncology, improving drug bioavailability and reducing side effects. The innovation presented in this study opens the door for broader applications of nanotechnology in cancer treatment.

Liu et al. reviewed diverse experimental models used in colorectal cancer (CRC) research, offering an in-depth overview of *in vitro* and *in vivo* models. Their work emphasizes the need to refine models to better replicate human CRC, providing a framework for testing targeted therapies like Shafiei’s MNNPs in ways that closely mimic human disease.

In hematological diseases, Li et al. examined the role of hemopexin (HPX) in mitigating heme toxicity during hemolysis, which commonly occurs in diseases such as sickle cell anemia and transfusion-induced hemolysis. While HPX protects against free heme, under certain conditions, it may exacerbate disease progression. This dual role emphasizes the need for more nuanced therapeutic approaches in hematology, similar to the precision seen in CRC treatments. Both studies demonstrate the complex dynamics of molecular precision in reducing harm and mitigating disease.

Zhang et al. presented a case study of a patient with severe combined immunodeficiency (SCID) who developed colon lymphoma due to a novel DCLRE1C mutation. It illustrates the intersection of immune system failure and cancer, highlighting the need for precision in both diagnostics and treatment. Early genetic diagnosis, as underscored by Li’s research on biomarkers, is critical for timely intervention, further emphasizing the importance of molecular precision in preventing and managing complex disease profiles.

Iacobescu et al. explored proteomics-based biomarkers for predicting and managing graft-versus-host disease (GVHD) in patients undergoing allogeneic hematopoietic stem cell transplantation (allo-HSCT). Their review delves into both single protein markers and protein panels, offering a comprehensive look at how these biomarkers can be used to stratify risk and diagnose GVHD. The authors emphasize how non-invasive biospecimens, such as blood or saliva, could be harnessed to enhance the precision of GVHD management.

Mocan et al. performed a proteomic exploration to identify novel serum biomarkers for intrahepatic cholangiocarcinoma (iCCA). Using high-throughput mass spectrometry, they identified several potential biomarkers, including S100A9, haptoglobin (HP), and serum amyloid A (SAA), which could distinguish iCCA from other liver conditions, such as cirrhosis and hepatocellular carcinoma. Their findings underscore the importance of proteomics in early cancer diagnosis and open the door to developing non-invasive diagnostic tools that could improve survival rates by enabling earlier detection and treatment.

Zhang et al. conducted a pan-cancer analysis of the TET family genes, particularly TET3, uncovering its role in tumor progression and drug sensitivity. Their bioinformatics analysis revealed that TET3 is involved in several cancer-related pathways, including cell cycle regulation, DNA damage response, and immune modulation. Targeting TET3 could inhibit tumor growth and increase chemotherapy sensitivity, positioning TET3 as a potential therapeutic target in a variety of cancers.

Chomczyk et al. provided an in-depth analysis of TP53 mutations in acute myeloid leukemia (AML) and their implications for disease progression. The review discussed the metabolic rewiring and immune evasion mechanisms driven by TP53 mutations, which are present in a subset of AML cases and associated with poor prognosis. Despite advances in targeted therapies, TP53-mutated AML remains a challenge due to its resistance to chemotherapy. This review called for further research into therapies targeting the metabolic vulnerabilities of TP53-mutated AML.

Munteanu et al. advanced the conversation around targeted therapies with their *in vivo* imaging system (IVIS) therapeutic assessment of tyrosine kinase inhibitor (TKI)-loaded gold nanoparticles for treating acute myeloid leukemia (AML). By using IVIS, the authors were able to track and assess the therapeutic effects of FLT3 inhibitors loaded into gold nanoparticles. Their preclinical study demonstrated that these nanocarriers improved drug delivery and bioavailability, offering enhanced tumor inhibition in FLT3-mutated AML models. This work reinforces the potential of nanomedicine in boosting the efficacy of precision therapies while minimizing systemic toxicity.

In conclusion, the studies featured in this Research Topic provide a comprehensive view of the current landscape of molecular targets in oncology and hematology. From novel drug delivery systems to biomarker identification, these contributions highlight the critical role of precision medicine in advancing treatment by targeting disease mechanisms, improving patient outcomes, and reducing the burden of traditional therapies.
